# Additional bone graft accelerates healing of clavicle non-unions and improves long-term results after 8.9 years: a retrospective study

**DOI:** 10.1186/s13018-014-0143-y

**Published:** 2015-01-09

**Authors:** Marc Schnetzke, Christian Morbitzer, Sara Aytac, Matthias Erhardt, Christian Frank, Matthias Muenzberg, Stefan Studier-Fischer, Lars Helbig, Arnold J Suda, Paul-Alfred Gruetzner, Thorsten Guehring

**Affiliations:** Department of Orthopaedic and Trauma Surgery, BG Unfallklinik Ludwigshafen, Ludwig Guttmann Strasse 13, 67071 Ludwigshafen am Rhein, Germany; Department of Surgery, Kreiskrankenhaus Grünstadt, Westring 55, 67269 Grünstadt, Germany; Department of Orthopaedic and Trauma Surgery, Klinikum Mittelbaden Baden-Baden Balg, Klinikum Mittelbaden GmbH, Balger Str. 50, 76532 Baden-Baden, Germany; Department of Orthopaedic Surgery, University of Heidelberg, Schlierbacher Landstraße 200a, 69118 Heidelberg, Germany

**Keywords:** Complicated fracture healing, Pseudarthrosis, Clavicle, Bone graft, Functional long-term outcome

## Abstract

**Background:**

Clavicle non-unions can occur after both conservative and operative treatment failure. Here, we investigated the outcome of patients with delayed fracture healing or non-unions of the clavicle. Patients underwent revision surgery by plate osteosynthesis of the clavicle with or without bone grafting. Our aim was to determine rates of bone healing and the functional long-term outcome.

**Methods:**

The study population of 58 consecutive patients was divided into group 1 (*n* = 25; no bone graft) and group 2 (*n* = 33; iliac crest bone graft). Bone consolidation was determined by the Lane-Sandhu score preoperatively and after 2.2 ± 1.8 years, respectively. The functional long-term outcome was determined after 8.9 ± 2.7 years in all available patients (*n* = 30) by the Constant score, DASH (Disabilities of the Arm, Shoulder and Hand) score and SF-36, and clavicle length was measured by ultrasound as compared to the healthy side.

**Results:**

Clavicle consolidation was achieved in 54 out of 58 patients (93.1%) after revision surgeries. The radiographic score and bone consolidation rates were significantly higher in group 2 (93.3%) as compared with 72% in group 1 (*p* = 0.02), resulting in a significantly shorter time to bone consolidation in group 2. Similarly, the relative risk for additional surgery after the first revision surgery was 4.7-fold higher in group 1 (*p* = 0.02). The long-term results showed overall very good results in DASH score (14.9 ± 16.5) and good results in Constant scores (77.9 ± 19.9). The group analyses found significantly better Constant scores and better visual analogue pain scale (VAS) numbers in group 2. Clavicle shortening appeared to affect the clinical results, and a mild correlation between shortening and Constant scores (*R* = −0.31) was found.

**Conclusions:**

This study shows high rates of bone healing and good functional outcomes after surgical revision of clavicle non-unions and further demonstrates that additional bone graft could significantly accelerate bone healing. This indicates that revision surgery of clavicle non-unions might preferably be done with additional bone graft, even if the surgeon considers that bone healing might be achieved without bone grafting.

## Background

Acute clavicle shaft fractures occur frequently and account for 2%–5% of all fractures [1,2], and there is still an ongoing debate on how to treat these fractures. The decision for treatment should be drawn by addressing the fracture site and the fracture stability; a relative consensus should exist for a conservative treatment to address stable, minimally displaced fractures of the clavicle shaft, while a surgical intervention may be required in cases of neurovascular compromise, open fractures, significant fracture displacement [3] or lateral fractures [4]. Generally, a trend in favour of a surgical therapy can be observed and includes plating using low-contact plates or minimally invasive intramedullary devices such as titanium elastic nails [5,6]. It should be noted that the type of surgical intervention should be drawn on an individual basis, and to date, no study has proven to affect outcome after fracture fixation [7]. In order to decide whether to treat clavicle fractures operatively or conservatively, a recent comprehensive review showed some evidence on the relative effectiveness of surgical versus conservative treatment for acute middle-third clavicle fractures [1], as related to an early decrease in pain, lower risk of mal-union and better functional outcomes [8,9].

Clavicle non-unions can occur after both operative and conservative treatments at a rate of 5%–6% [10], and there is some evidence that non-union rates are lower after surgery [1,11].

Risk factors for non-union include a clavicle shortening of >2 cm [12], displaced and unstable lateral Neer type II fractures [4] and particularly in combination with initial severe soft tissue trauma [8].

Once a clavicle non-union has developed, Jupiter and Leffert described the current gold standard treatment of plating *with* or *without* bone grafting [13]. All further studies addressing this concept found good bone healing results in general [14-17]. However, some authors questioned the need of distant bone grafts from the iliac crest and suggested plating alone [18], especially in hypertrophic types of non-unions [16]. Similarly, Endrizzi et al. concluded that a bone graft might not be necessary in most cases of clavicle non-union [19]. Therefore, based on the current literature, bone grafting may not be necessary *in every case*. It is recommended that bone graft should be used individualized and adapted to patient and non-union characteristics [8].

Thus, the aim of the current study was to find out whether plating with local bone preparation or alternatively with additional bone graft from the iliac crest influenced bone healing rates in patients with clavicle non-unions or delayed fracture healing. A secondary aim was to find out whether long-term functional results were affected by bone grafting and whether clavicle length restoration might have influenced the results.

## Methods

### Study population and treatment groups

**Figure 1:**
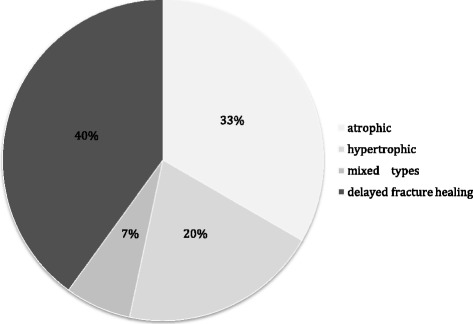
**Study population with variation of the included types of fracture healing complication.**

This retrospective study obtained the local ethical review committee approval and included 58 consecutive patients with clavicle non-unions (>6 months after injury) or delayed fracture healing (<6 months after injury) [20]. The distribution of hypertrophic and atrophic types of non-unions [21] is illustrated in Figure 1.

All patients were treated at a level 1 trauma centre from 2001 to 2009. Patient mean age was 38.7 ± 12.4 years at injury (range 19–67). The study included 16 female and 42 male patients. Relevant comorbidities included coronary heart disease (*n* = 2), hypertension (*n* = 12), chronic airway diseases (*n* = 7) and low back pain (*n* = 18), and their distribution showed that 56.7% of patients had >2 comorbidities, while 16% had no comorbidity. No patients had diabetes or malignancies; 46.7% of all patients were smokers.

Initial treatment before revision surgery was conservative in 38 patients (65.5%) and operative in 20 patients (34.5%; intramedullary or plate fixation); 12.1% of patients had a polytrauma, and 31% had several fractures. The initial injury mechanisms were sports accidents, traffic accidents or falls from a height.

The time between injury and revision surgery was 12.5 ± 14.4 months (group 1: 11.1 ± 12.0; group 2 13.4 ± 16.2).

**Figure 2:**
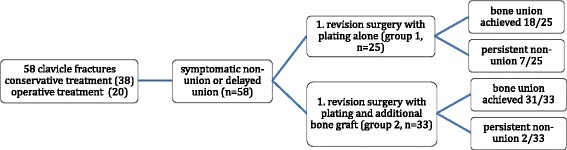
**Flowchart with an overview of patient selection and treatment groups.**

All patients (*n* = 58) underwent plating of the clavicle during revision surgery and were subdivided into two treatment groups: group 1 (*n* = 25 patients, 43.1%) received a local clavicle bone preparation with removal of the bone sclerosis at the site of non-union. Group 2 (*n* = 33 patients, 56.9%) received bone graft from the iliac crest (tricortical block or cancellous bone) in addition to a local preparation. A flowchart of patient selection and treatment is shown in Figure 2. The distribution of patients with hypertrophic and atrophic non-unions was similar in both groups.

### Indication and technique of revision surgery

**Figure 3:**
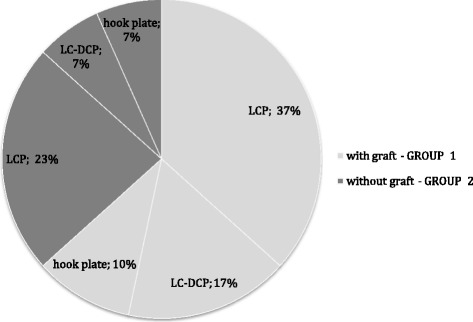
**Distribution of implant types during revision surgery.**

Symptomatic non-union or delayed fracture healing [20] (atrophic or hypertrophic forms according to [21]) with instability and pain indicated revision surgery. Inclusion and exclusion criteria are shown in Table 1. No patient had neurovascular symptoms. Experienced attending trauma surgeons performed all surgeries. Plates of variable length were applied to the superior clavicle surface. The following implants were used: locking compression plate (LCP; 60%; Synthes, Umkirch, Germany), limited contact dynamic compression plates (LC-DCP; 23%; Synthes, Umkirch, Germany) and hook plates of the lateral clavicle shaft (17%, Synthes, Umkirch, Germany) (Figure 3). During revision surgery, the non-union was compressed by using the AO compression device or lag screws. If the lag screws were inserted via the non-union, they were removed after the plate was fixed. Intraoperatively, the surgeon decided whether a distant bone graft from the iliac crest was used in addition to petalling [22] and a local preparation of clavicle ends (group 2). A single-shot antibiotic was administered after cultures were taken from the non-union. In accordance to our algorithm for the use of distant bone grafts, reasons not to use distant bone graft in group 1 were predominant hypertrophic forms of delayed union or non-union and well-vascularized bone beds after local preparation. In the case of larger bone defects with clavicle shortening, a tricortical bone was harvested from the iliac crest, whereas cancellous bone was used in smaller defects. In the case of a further revision surgery after failed bone consolidation, patients always underwent bone grafting. The rehabilitation was similar in both groups and included physiotherapy with a 90° limited abduction of the shoulder for 6 weeks.

**Table 1:** **Inclusion and exclusion criteria**

**Inclusion criteria**	**Exclusion criteria**
Age >18 years Symptomatic delayed or non-union after clavicle fracture Informed consent	Osteitis/positive intraoperative cultures after primary treatment Malignancy Metabolic bone disease No informed consent

### Clinical and radiographic outcome parameters with long-term follow-up

Radiographic results and bone healing rates were determined in all 58 patients in the latest X-rays. The radiographs were reviewed by two independent observers (MS and TG). Bone consolidation was determined in preoperative and postoperative X-rays, and to determine the time to bone fusion, all available X-rays were reviewed for bone consolidation. For radiographic outcome and bone healing, the Lane-Sandhu scoring system [14] was applied in both groups (Table 2). Postoperative radiographic scores were compared to preoperative numbers. Rates of surgery-associated complications with required re-osteosynthesis were determined.

**Table 2:** **Lane-Sandhu scoring system**

**Score**	**Radiologic findings**
0	No callus
1	Minimal callus
2	Callus evident but healing incomplete
3	Callus evident with stability expected
4	Complete healing with bone remodelling

The long-term results after 8.9 ± 2.7 (5–13) years were reported in 30/58 patients (51.7%). Most patients were lost due to change of residency. Clinical outcome was determined by questionnaires (DASH (Disabilities of the Arm, Shoulder and Hand), Constant, visual analogue pain scale (VAS), SF-36). Preoperative VAS numbers were taken from the patient’s records. Additional requested items included required implant removal and return to previous work and daily (sports) activity. The length of the injured clavicle was compared with the non-injured side. An experienced examiner measured the length after identifying the sterno-clavicular and acromio-clavicular joint lines by ultrasound control.

### Statistics

Mean and standard deviation (SD) were calculated for continuous variables, and mean and median were calculated for ordinal variables. The primary outcome parameter was the Constant score. Differences between the Constant score and a variable were tested by Student’s *t* in the case of data with normal distribution. The chi-square test was used in the analysis of contingency tables. A correlation between two variables was calculated by Pearson’s coefficient *R*.

## Results

### Bone healing after the first and further revision surgeries and radiographic results (*n* = 58)

Average radiologic follow-up was 2.2 ± 1.8 years in all patients (Table 3). Overall bone fusion could be achieved in 54/58 patients (93.1%). Due to persisting non-union after the first revision surgery, a further re-osteosynthesis was required in nine patients after 5.6 ± 4.9 months, seven in group 1 and two in group 2. This led to significantly higher revision rates in group 1 (7/25 patients; 28%) than in group 2 (2/33 patients; 6%; *p* = 0.02) and a 4.7 higher relative risk (RR) of additional surgery in group 1 (Table 4). During additional surgical revision, all nine patients with failed bone fusion after the first revision surgery were treated with bone grafting by a tricortical bone block. Four patients (three from group 1) achieved no sufficient bone consolidation even after the second revision, and two patients achieved no union after a total of four revisions. Preoperatively, no significant difference was found in Lane-Sandhu scores of groups 1 and 2. The Lane-Sandhu score improved from preoperative numbers to higher numbers after the first revision surgery, though a significantly higher Lane-Sandhu score was found in group 2 (3.7 ± 1.0), compared to group 1 (2.6 ± 1.8) (*p* = 0.001). In group 2, eight patients with larger bone defects received a tricortical bone graft, while 25 patients obtained a cancellous bone transplantation. Interestingly, this did not affect bone healing as determined by the Lane-Sandhu score, nor did it affect clavicle shortening and Constant scores (see below).

**Table 3:** **Clinical and radiological follow-up**

	**Group 1 (** ***n*** **= 25)**	**Group 2 (** ***n*** **= 33)**	***p*** **value**
Clinical follow-up (years)	8.8 ± 2.4	9.0 ± 3.0	0.88
Radiologic follow-up (years)	2.5 ± 2.2	2.0 ± 1.5	0.18

**Table 4:** **Fusion and revision rates in both groups**

	**Group 1 (** ***n*** **= 25)**	**Group 2 (** ***n*** **= 33)**	***p*** **value**
Bone consolidation after the first revision	18/25 (72%)	31/33 (93.9%)	0.02
Bone consolidation after all revisions	22/25 (88%)	32/33 (97%)	0.18
Lane-Sandhu score preoperatively	0.48 ± 0.71	0.6 ± 073	0.34
Lane-Sandhu score after the first revision	2.6 ± 1.8	3.7 ± 1.0	0.001
Lane-Sandhu score after all revision	3.4 ± 1.4	3.8 ± 0.7	0.21
Further revisions (no. of patients/no. of surgeries)	7/9	2/5	0.02
Time to bone consolidation (months)	10.3 ± 9.5	4.7 ± 3.4	0.02

### Clinical long-term outcome (*n* = 30, group 1: *n* = 11; group 2: *n* = 19)

**Figure 4:**
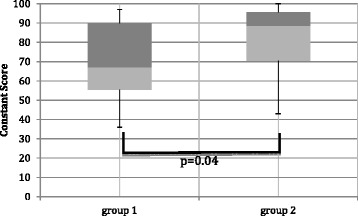
**Results of the Constant score after 8.9 ± 2.7 years, with variation of treatment groups;** ***p*** **= 0.04.**

After 8.9 ± 2.7 years, the Constant scores turned out significantly better in group 2 with 82.7 ± 16.9 vs. 69.2 ± 58.9 in group 1 (*p* = 0.04; Figure 4). Similarly, the DASH score showed considerably better results in group 2, though without statistical significance (11.7 ± 13.3 vs. 21.4 ± 21.1; *p* = 0.07). The scores from SF-36 showed overall good results irrespective of groups; the sub-item “physical functioning score” showed significantly better results in group 2 (group 2: 85.8 ± 15.7; group 1: 68.0 ± 32.1; *p* = 0.049).

No significant differences in Constant scores were found by variation of plate type (LCP: 76.7 ± 21.6; LC-DCP: 83.6 ± 14.4; hook plate: 72.0 ± 24.6, comparison between plate types *p* > 0.05), anatomical site of the clavicle non-union (mid-shaft: 78.6 ± 19.8; lateral: 72.0 ± 21.6; *p* = 0.60), initial conservative or operative treatment (*p* = 0.63), type of fracture healing complication (delayed fracture healing vs. non-union; *p* = 0.90) or smoking (*p* = 0.61). Two and more comorbidities affected the outcome, i.e. Constant scores in patients with at most one comorbidity were 92.3 ± 6.1 compared to 64.4 ± 22.2 in patients with two or more comorbidities (*p* = 0.0003). It should be noted that variables such as comorbidities, type of plate and type of complication were evenly distributed in both treatment groups.

VAS improved significantly in both groups between preoperative evaluation and time of discharge, with a further decrease with time. In agreement with Constant scores, patients from group 2 had significantly less pain after 8.9 years (*p* = 0.04) (Table 5).

**Table 5:** **Overview of functional long-term results**

**Score**	**Group 1 (** ***n*** **= 25)**	**Group 2 (** ***n*** **= 33)**	***p*** **value**
VAS preoperatively	5.8 ± 2.1	6.3 ± 3.3	0.65
VAS at discharge	2.9 ± 1.9	3.6 ± 3.0	0.50
VAS after 8.9 years	1.9 ± 2.4	0.5 ± 1.0	0.04
Constant score after 8.9 years	69.2 ± 58.9	82.7 ± 16.9	0.04
DASH score after 8.9 years	21.5 ± 21.1	11.7 ± 13.3	0.07
SF-36 (physical functioning score)	68.0 ± 32.1	85.8 ± 15.7	0.049

**Figure 5:**
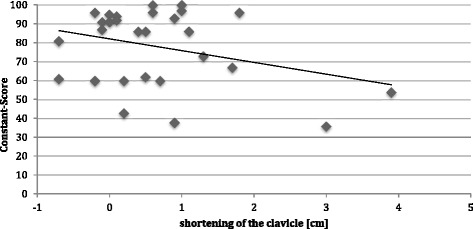
**Correlation between shortening of the clavicle and Constant score, irrespective of the treatment group.**

**Figure 6:**
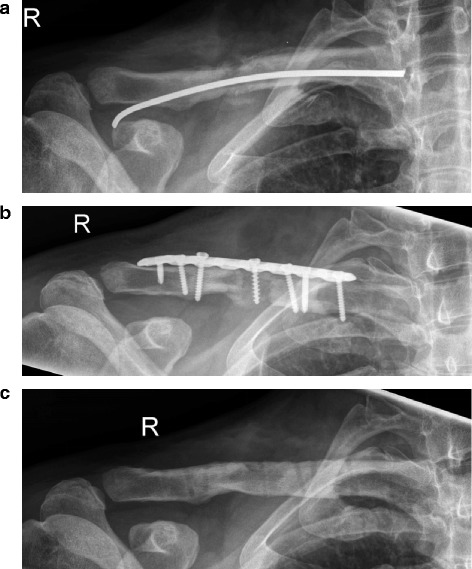
**Non-union (right-sided, R) 6 months after fixation with a titanium elastic nail in a 43-year-old male with mechanical instability (a-c). (b)** Result after revision with distant bone graft, length correction and LCP fixation. **(c)** Bone consolidation after metal removal.

Length measurements by ultrasound of injured versus healthy clavicles showed less shortening of the clavicle in group 2 (0.47 ± 0.65 vs. 0.95 ± 1.4 cm; *p* = 0.10). A correlation analysis demonstrated only a mild negative correlation between clavicle length and Constant scores in all patients (*R* = −0.31; Figure 5), indicating that shortening of the clavicle was slightly associated with lower Constant scores. While two patients with a clavicle shortening of >3 cm showed a considerably reduced range of motion, no significant correlation between range of motion and clavicle shortening was found in all patients. In order to further investigate the association of clavicle length and clinical results, five patients with a clavicle shortening of >1 cm were identified and showed a significantly lower Constant score (70.0 ± 19.1 vs. 82.4 ± 17.0; *p* = 0.036). A treatment example with lengthening of the clavicle by a tricortical block is shown in Figure 6a,b,c.

In group 2, the reconstruction of the clavicle by cancellous bone or tricortical block, respectively, did not influence the outcome of clavicle length and Constant scores.

All patients returned to previous work; 8/11 patients (72.7%) in group 1 returned to previous sports activity after 16.4 ± 10.7 weeks, compared with 15/19 patients (78.9%) in group 2 after 30.5 ± 52.7 weeks (*p* = 0.57).

Implant removal was done in 34 patients after an average of 20.7 months after revision surgery (group 1: 21.7 months, 10/25 patients (40%); group 2: 20.5 months, 24/33 patients (72.7%)).

### Treatment failures and complications

Nine out of 58 patients (15.5%) required further revision surgery after the first revision (see above). Four patients (6.8%) did not achieve bone consolidation during follow-up and developed an infection-related non-union, which was relatively asymptomatic.

After grafting, 7/33 patients (21.2%) reported temporary pain at the donor site as a minor complication, which was resolved soon.

## Discussion

In this study, we show high rates of bone fusion (>93%) with good long-term functional outcomes in DASH and Constant scores after revision surgery in patients with clavicle non-unions. Interestingly, after comparing patients treated *with* or *without* bone grafts, we found significantly higher rates of treatment failures after the *first* revision surgery in group 1 without bone graft (28% in group 1 vs. 6% in group 2; RR = 4.7), requiring additional revision surgery *with* iliac bone graft. This led to a significantly shorter time to bone consolidation in patients treated with additional bone graft (group 2). Moreover, patients from group 1 had worse long-term functional results with higher pain levels and lower Constant scores (*p* = 0.04; Figure 4, Table 5).

The lower bone healing rates in group 1 with a 4.7-fold higher risk of additional surgery indicate that bone grafts should be used, even if the surgeon might consider that a distant bone graft is not necessary due to reasons such as hypertrophic non-unions or delayed unions with sufficient vasculature at the fracture ends. To our knowledge, this is the first study that compared bone healing rates *with* or *without* additional bone graft in patients with clavicle non-unions. Although separation into treatment groups could be biased and influenced by the surgeon’s individual opinion, it should be noted that the treatment groups did not show unequal distribution of confounders such as type of bone healing complication or comorbidities.

One other study evaluated the need for distant bone graft in comparison with local bone preparation. Only three of 43 patients obtained distant bone grafts, while 30 underwent local bone preparation and 14 received a demineralized bone matrix. Good clavicle consolidation rates in this study led to the conclusion that distant bone grafts might not be necessary *in most cases* [19].

The current study results did not agree with reports by Jubel et al. showing successful healing in 14 patients after re-osteosynthesis using titanium elastic nails *without* distant bone graft [23]. Similarly, Baker and Mullett found successful bone union after revision plating *without* bone graft in 14 initially conservatively treated patients, indicating that bone graft might not be necessary in the case of adequate local bone preparation and stable fixation [18].

A number of reports support our current study findings. Faraud et al. came to the conclusion that treatment of middle-third clavicle non-union after initial failure of conservative treatment with stable fixation and bone graft is a reliable, well-suited and effective treatment [24]. Jupiter and Leffert successfully used bone grafts in 18 of 21 patients [13], concluding that surgical revisions should include iliac crest bone by utilizing its osteogenic, osteoconductive and osteoinductive properties [25,26]. Collinge et al. suggested bone grafts in any case of clavicle non-union [15]. Similarly, Khan et al. used LCPs *with* bone graft in patients with poor bone stock and reported fusion in 11 patients after 2.8 years with good functional outcome [16]. Similarly to our current study findings, Laursen and Dossing yielded a high rate of healing and an acceptable functional outcome in patients with clavicular non-unions treated with compression plate and autologous cancellous bone graft [27]. All these studies strengthen the hypothesis of our study that bone grafting of clavicle non-unions is important to improve bone healing rates.

Olsen et al. performed a surgical revision by restoration of the clavicle length by bone graft and found healing in 15 of 16 patients [26]. Similarly, O’Connor et al. reported bone healing in 22 of 24 patients after distant bone graft, though with higher DASH scores [25]. A recent study emphasized clavicle length correction by a tricortical iliac crest graft and LCP stabilization [17].

Very limited data is available on functional long-term results of clavicle non-unions. Bradbury et al. reported bone healing after bone graft in 31/32 patients with a Constant score of 87 after 8 years [14]. O’Connor et al. followed patients throughout 42 months [25], while all other studies reported a longest follow-up period of 2.8 years [16]. Thus, the mean follow-up of 8.9 years in the current study appears appropriate to evaluate the long-term outcome.

Patients with additional bone graft showed improved functional results after 8.9 years, with better Constant scores, lower pain and similar trends for SF-36 and DASH scores (Figure 4, Table 5). These data indicate that bone grafts not only increase bone healing rates during initial clavicle non-union revision surgery but also might lead to a better long-term functional outcome. The mechanisms might be related to a better restoration of the correct length of the injured clavicle, as shown by a mild inverse correlation between clavicle shortening and Constant scores (Figure 5) and supported by the fact that five patients with a shortening of more than 1 cm had significantly lower Constant scores. Clavicle shortening might lead to a glenoid malposition with abduction and overhead motion deficits [28].

We used the current gold standard of superior clavicle plate fixation [13], including modern anatomical locking screws [17], although studies suggested alternative fixation, i.e. by intramedullary K-wires, titanium elastic nails [23,29], screws [30] or pinning [31]. We used hook plates, LCPs and LC-DCPs to achieve stable fixation of the clavicle. No statistical differences were found between the different types of plates. The type of non-union (hypertrophic, atrophic or a mixture of both types [21]) did not affect the outcome. Similarly, Lai et al. found no significant difference in outcome between LCPs and LC-DCPs in acute mid-shaft fractures [32]. We used different types of plates without differences in outcome but did not investigate different plate positions. One advantage of the anterior-inferior plate positioning includes a less prominent implant position [33]. In the current study, this assumption might explain the high rate of metal removal after bone consolidation (58.6%) due to local irritation.

Despite the retrospective set-up, a clear strength of this study is the long-term functional outcome and comparison of the two different treatment groups. To our knowledge, this study is the first that compares plating alone and plating with bone grafting in clavicle non-union. Limitations included the shortcomings inherent to the retrospective set-up of this study. Another limitation includes the fact that the decision for distant bone graft was guided by the surgeon’s personal experience (see previous paragraph), which might bias the results of this study. Moreover, we lost considerably a high amount of patients during the long follow-up period, compared to other studies [19]. It should be noted that the current study results should be verified in further prospective studies.

## Conclusion

In light of the current retrospective findings, we have adopted our revision strategy. Despite individual pros and contras associated with patient and non-union characteristics, we rather tend to use additional bone graft even in patients with delayed fracture healing and hypertrophic non-unions. The amount of transplanted bone should be adjusted—from small amounts to tricortical blocks—depending on the defect size after resection of the non-union and clavicle shortening.

## Abbreviations

LCP: Locking compression plate
LC-DCP: Limited contact dynamic compression plates
